# Multivariate genomic and transcriptomic determinants of imaging-derived personalized therapeutic needs in Parkinson’s disease

**DOI:** 10.1038/s41598-022-09506-0

**Published:** 2022-03-31

**Authors:** Christophe Lenglos, Sue-Jin Lin, Yashar Zeighami, Tobias R. Baumeister, Felix Carbonell, Yasser Iturria-Medina

**Affiliations:** 1 Neurology and Neurosurgery Department, Montreal Neurological Institute, McGill University, 3801 University Street, Room NW312, Montreal, H3A 2B4 Canada; 2 McConnell Brain Imaging Centre, Montreal Neurological Institute, McGill University, Montreal, Canada; 3 Ludmer Centre for Neuroinformatics and Mental Health, McGill University, Montreal, Canada; 4 Biospective Inc., Montreal, Canada

**Keywords:** Parkinson's disease, Biomarkers, Genetics research, Computational models

## Abstract

Due to the marked interpersonal neuropathologic and clinical heterogeneity of Parkinson's disease (PD), current interventions are not personalized and fail to benefit all patients. Furthermore, we continue to lack well-established methods and clinical tests to tailor interventions at the individual level in PD. Here, we identify the genetic determinants of individual-tailored treatment needs derived from longitudinal multimodal neuroimaging data in 294 PD patients (PPMI data). Advanced multivariate statistical analysis revealed that both genomic and blood transcriptomic data significantly explain (*P* < 0.01, FWE-corrected) the interindividual variability in therapeutic needs associated with dopaminergic, functional, and structural brain reorganization. We confirmed a high overlap between the identified highly predictive molecular pathways and determinants of levodopa clinical responsiveness, including well-known (Wnt signaling, angiogenesis, dopaminergic activity) and recently discovered (immune markers, gonadotropin-releasing hormone receptor) pathways/components. In addition, the observed strong correspondence between the identified genomic and baseline-transcriptomic determinants of treatment needs/response supports the genome's active role at the time of patient evaluation (i.e., beyond individual genetic predispositions at birth). This study paves the way for effectively combining genomic, transcriptomic and neuroimaging data for implementing successful individually tailored interventions in PD and extending our pathogenetic understanding of this multifactorial and heterogeneous disorder.

## Introduction

Parkinson’s disease (PD) is a complex disorder with abnormalities including synucleinopathy (misfolded alpha-synuclein aggregates), dopaminergic neuronal loss, brain functional dysregulation and structural atrophy^1^. Due to the marked interpersonal variability/heterogeneity and multifactorial nature of PD and related disorders, numerous treatments targeting only one of these biological components have failed to achieve significant clinical benefits at the population level^1,2^. To succeed, interventions may instead focus on individual therapeutic needs and combinations of treatments targeting multiple components, as proposed by the *personalized medicine* approach^3–7^. Unfortunately, we currently do not have well-established methods or clinical tests to tailor interventions at the personalized level across several biological factors in PD. This gap relates to critical methodological limitations, such as the requirement for an understanding of the causal disease mechanisms and the inability to predict an individual's brain response to different therapeutic interventions.

Motivated by the lack of a clear understanding of PD’s intrinsic complexity and heterogeneous response to treatment, recent approaches have adopted a multifactorial perspective^2,3^. Neuroimaging data and/or novel machine learning techniques have provided promising results for predicting individual responsiveness to levodopa^4^, deep brain stimulation (DBS^5^) and stem cell transplant^6^. These independently proposed approaches, however, have focused on identifying unique predictive signatures for each specific treatment. While the identified biomarkers provide information about the potential individual response to a given therapeutic strategy, they fail to clarify whether other alternative treatments require similar (or not) patient conditions. With this in view, detecting portable and generalizable predictors of personalized therapeutic needs will significantly improve clinical decisions with regards to not only one but also multiple potential treatment strategies. For instance, in the Alzheimer’s disease (AD) context, a personalized therapeutic intervention fingerprint (pTIF) has been recently proposed^7^. pTIF constitutes a simplified individual patient profile of the quantitative biological factor modifications needed to control disease evolution. It assumes that the patients may need different treatments, depending not only on their brain's unifactorial alterations/biomarkers (e.g., dopamine alteration or not, atrophy or not) but also on their individual multifactorial brain dynamics: how the different biological factors interact and how they could respond (at the individual level) to potential clinical perturbations^7^. Based on spatiotemporal analysis of multimodal imaging data (i.e., positron emission tomography (PET), magnetic resonance imaging (MRI), single photon emission computed tomography (SPECT)), pTIF values are a set of multivariate metrics that reflect the biological reformation required to stop the pathologic progression or revert the condition to normality. The results in late-onset AD support the notion that pTIF allows the categorization of patients into distinctive therapeutic-based subtypes, with patients in the same pTIF subtype presenting a distinctive pattern of molecular alterations^7^.

On the other hand, detecting genes with the capacity to modify response to treatment will significantly improve clinical interventions by identifying subjects that can benefit from therapy and those at an increased risk of harm^8–10^. Pharmacogenetics aims to identify patients at higher genetically determined risk of drug adverse effects or ineffective medication to modify dosage or switch to an alternative therapy^11^. Numerous susceptibility loci have been reported in terms of both levodopa treatment efficacy and adverse responses, and most of the identified genes are related to the dopaminergic pathway^12^. However, recent genetic examinations of treatment responses in PD patients have almost exclusively been predicated on univariate analyses^13^. Based on traditional genome-wide association studies (GWAS) that identify single risk-related single-nucleotide polymorphisms (SNPs) or loci, these approaches fail to analyze multiple clinical traits/aspects at the same time and, importantly, are unable by definition to discover clusters of functionally related genes and pathways^13^. Furthermore, the field has uniformly focused on a single treatment option (levodopa), while the molecular basis of individual predisposition to other potential therapeutic strategies remains unexplored.

Prompted by the imperative for identifying effective individually tailored treatments in PD, we extensively investigated the genetic and multifactorial brain basis of different treatment needs and responsiveness in this disorder. First, we aimed to discover the causal genetic determinants of personalized treatment needs derived from multimodal neuroimaging data, reflective of the brain’s complex reorganization process and potential response to different treatments^7^. Subsequently, to assess the portability of the identified treatment needs and their genetic determinants, we aimed to clarify whether similar molecular mechanisms would modulate clinical outcomes to levodopa. By concurrently analyzing several imaging features or clinical variables as interrelated genome-dependent factors, novel multivariate statistical analyses identified causal genetic effects on treatment requirements and observed responsiveness in PD in the presence of potentially pleiotropic and correlated genes. A strong molecular-based overlap between brain imaging-estimated multifactorial therapeutic needs and clinical response to levodopa was found, having in common highly predictive molecular pathways. Shared genetic-neuroimaging and genetic-clinical functional pathways included well-known PD-associated molecular functions (e.g., angiogenesis, modulation of dopaminergic cell activity) and more recently proposed PD mechanisms (e.g., gonadotropin-releasing hormone receptor, immune markers). Furthermore, a complementary analysis with blood gene expression (GE) confirmed the active role of the identified genomic-based molecular pathways at the time of patient evaluation. In addition to extending the pathogenetic understanding of this complex disease from a multivariate integrative perspective, our findings support the crucial need for extending the combined interrogation of genomic and neuroimaging data in pursuit of effective individually tailored treatments in PD.

## Results

**Figure 1:**
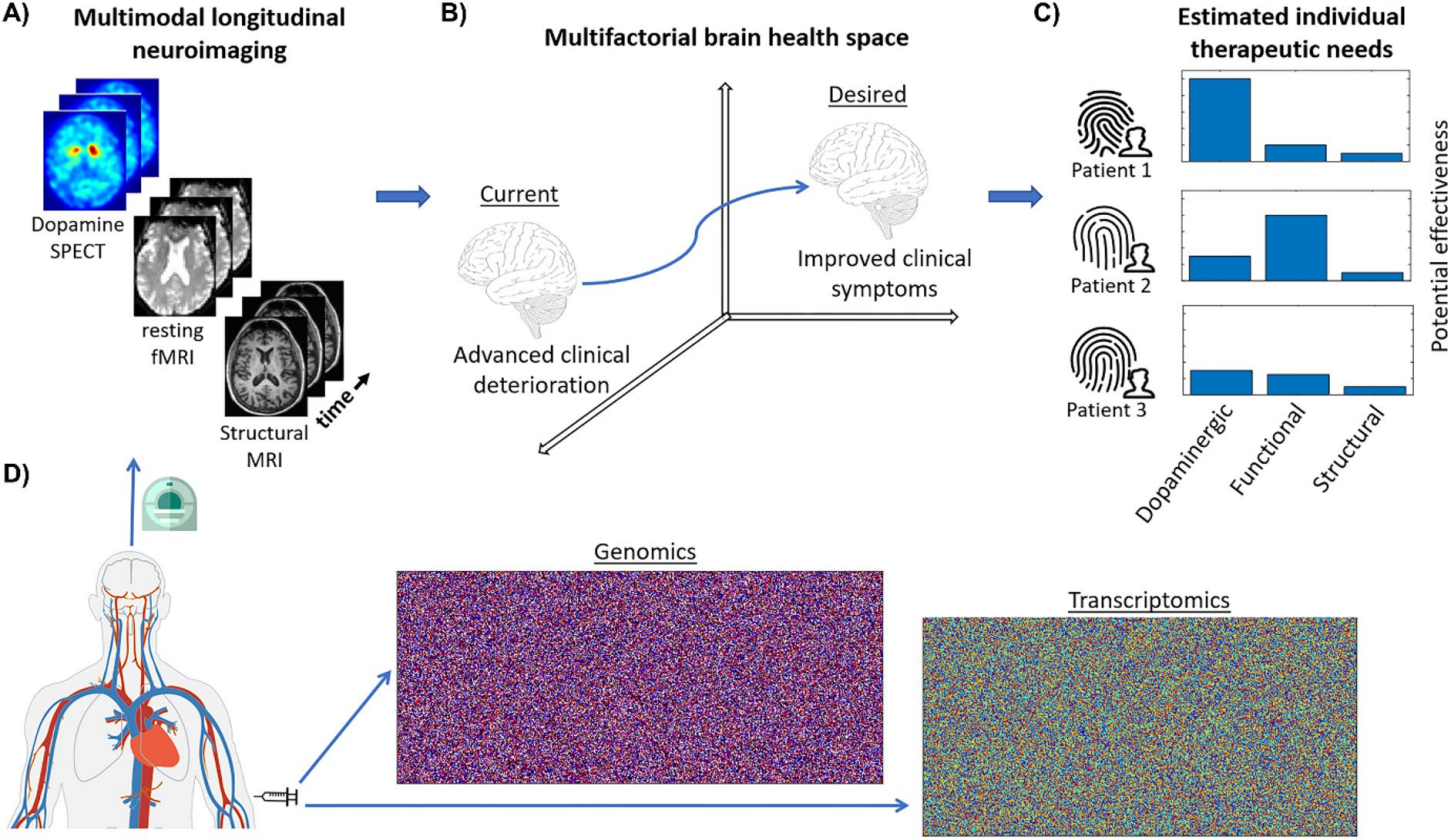
Workflow for multifactorial therapeutic intervention fingerprinting in PD. (**A**) Longitudinal imaging for dopamine SPECT, functional MRI at rest and/or structural MRI. (**B**) A network-based approach^7^ allows individual characterization of intra-brain synergistic biological interactions and multifactorial spreading mechanisms through anatomical connections. Inverting the model’s fundamental equation allows estimation of the changes required to produce a desired clinical effect (i.e., conducing the patient’s brain from the current neurodegenerative state towards a healthier clinical condition). (**C**) Dissimilar pTIF patterns for three participants with the same diagnosis. For each patient, the pTIF is defined as the set of biological changes required, estimating how clinically effective it would be to target each analyzed biological process. In this example, note that Patient 1 would be more benefitted by a dopamine-based therapeutic intervention (e.g., Levodopa treatment), while for Patient 2 it would be more effective a functional intervention (e.g. Deep brain stimulation or Transcranial magnetic stimulation), suggesting the identification of specific single-target therapies that may benefit these patients. However, Patient 3 may not be clinically benefitted by any of these three single-target interventions, suggesting that combinatorial (and not single-target) treatments would be more appropriate in this case. For visual simplicity, in this figure only single-target interventions are represented, but for three neuroimaging modalities the pTIF includes 7 global values, corresponding to each modality and their combinations (see “Material and methods”, “Imaging-derived individual therapeutic needs estimation”). (**D**) Genomic and Transcriptomic data is collected for identifying the genetic basis of the estimated treatment needs in PD.

Multimodal longitudinal neuroimaging data, genetic (DNA, RNA), baseline clinical evaluations, demographics and/or medication data were collected for 294 PD patients (see Fig. S1 for flowchart of participants selection and analysis). Three modalities of multimodal imaging were longitudinally assessed: structural T1-MRI (for quantifying gray matter density), functional-MRI at rest (for functional integrity) and/or DatSCAN SPECT (for dopaminergic neuronal loss). Recently, we proposed a neuroimaging-derived personalized fingerprinting (pTIF) method for predicting individual therapeutic needs based on the quantification of the brain’s multifactorial reorganization (see Fig. 1; “Material and methods”, “Imaging-derived individual therapeutic needs estimation”). Here, we used this approach^7^ to estimate the therapeutic needs in PD subjects and subsequently determine their associated genetic determinants. For the 294 PD patients, 7 unique elements of global pTIFs were obtained based on the individual longitudinal dopaminergic, functional, and structural imaging-inferred measurements and their combinations. Note that the number 7 of pTIF elements corresponds to all possible single-target or combinatorial interventions focused on the three biological factors quantified by the used imaging modalities. Specifically, these 7 numerical values reflect the required whole-brain reformations for targeting dopaminergic integrity (DOP), functional activity at rest (FUNC), gray matter (GM), and the combinations GM-FUNC, GM-DOP, FUNC-DOP, and GM-FUNC-DOP (see Fig. 1, and “Material and methods”).

**Figure 2:**
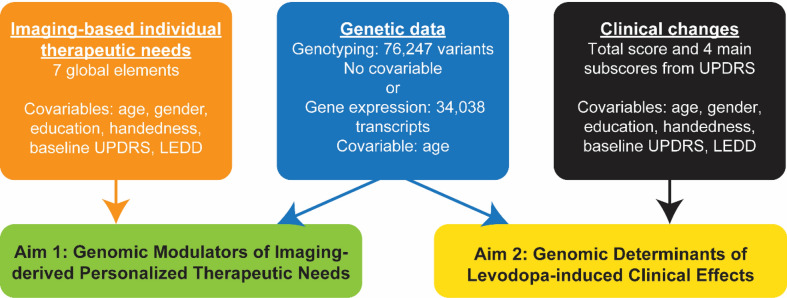
Schematics for determining causal genetic determinants of individual therapeutic needs and treatment response in PD. Two main analyses are performed: PLS-SVD on genetic data vs imaging-based individual therapeutic needs and genetic data vs clinical changes. [UPDRS = the Unified Parkinson's Disease Rating Scale part III (ON medication), LEDD = levodopa equivalent daily dose].

Genetics included genome-wide genotyping of 76,247 SNPs and abundance levels for 34,038 gene transcripts. Longitudinal clinical evaluations (available for 216 patients) consisted of the Movement Disorder Society-Unified Parkinson's Disease Rating Scale (MDS-UPDRS) motor part (4 main subscores and a total score; see Fig. 2; “Material and methods”).

### Genomic modulators of imaging-derived individual therapeutic needs in PD

First, we aimed to identify potential causal genotyping determinants of the imaging-derive individual therapeutic needs in PD patients (Fig. 2, Aim 1). To statistically consider the high dimensionality and autocorrelation of the genomic data, we used a data-driven multivariate cross-correlation analysis in combination with randomized permutation and bootstrapping tests (“Material and methods”, “Multivariate statistical analyses”). Multiple potential confounders in the pTIF estimations were included (i.e., age, sex, educational level, handedness, baseline MDS-UPDRS motor score and levodopa equivalent daily dose [LEDD]). By concurrently analyzing changes in several imaging-derived variables (7 global pTIFs), this multivariate analysis searched for large clusters of functionally related SNPs that were statistically associated with the imaging outputs in the presence of potentially pleiotropic and correlated genes. In practice, the applied singular value decomposition (SVD) method focused on identifying the specific set of gene variants maximally related to therapeutic needs while controlling for covariates. This SVD^14^ generalizes both principal component analysis (PCA)^15^ and partial least squares (PLS)^16^.

**Figure 3:**
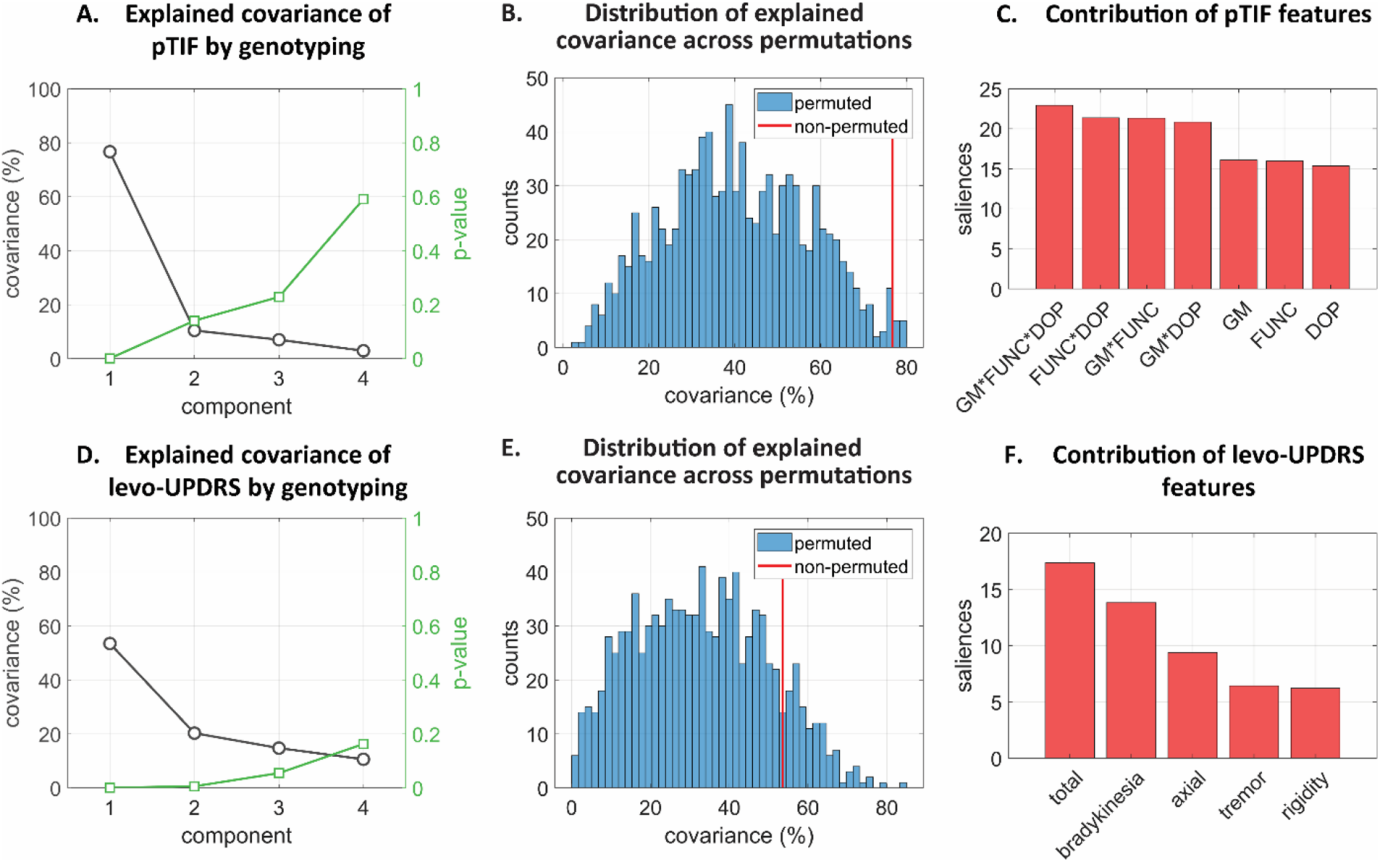
Multivariate cross-correlation results between genotyping and imaging-derived therapeutic needs. (**A**) Explained covariance of pTIF by genotyping for each principal component. (**B**) Distribution of explained covariance across randomized permutations for the first significant principal component (PC1). This was used to calculate the cross-validated added explained variance (the added value was defined as the difference between the original pTIF-genomics shared variance with the mean value of the randomized distribution). (**C**) Contribution of pTIF features in PC1. (**D**) Explained covariance of levodopa-induced UPDRS changes (levo-UPDRS) by genotyping for the obtained principal components. (**E**) Distribution of explained covariance across permutations for PC1. (**F**) Contribution of levo-UPDRS features in PC1.

We observed (Fig. 3) that the genetic variants shared a high covariance with the global pTIF elements, with the first significant principal component (PC1) of the genomic data explaining up to 76.70% of the pTIF population variance (*P* = 0.001, familywise error (FWE)-corrected; cross-validated added explained variance of 36%). Additionally, to ensure that race and Levodopa medication during fMRI did not bias these results, we repeated the analysis with patients with only white ancestry and on off-medication during fMRI acquisition, respectively. In both cases (Fig. S4), a strong similarity/consistency with the whole-population results were observed, confirming our findings’ stability across different races and potential medication effects.

**Figure 4:**
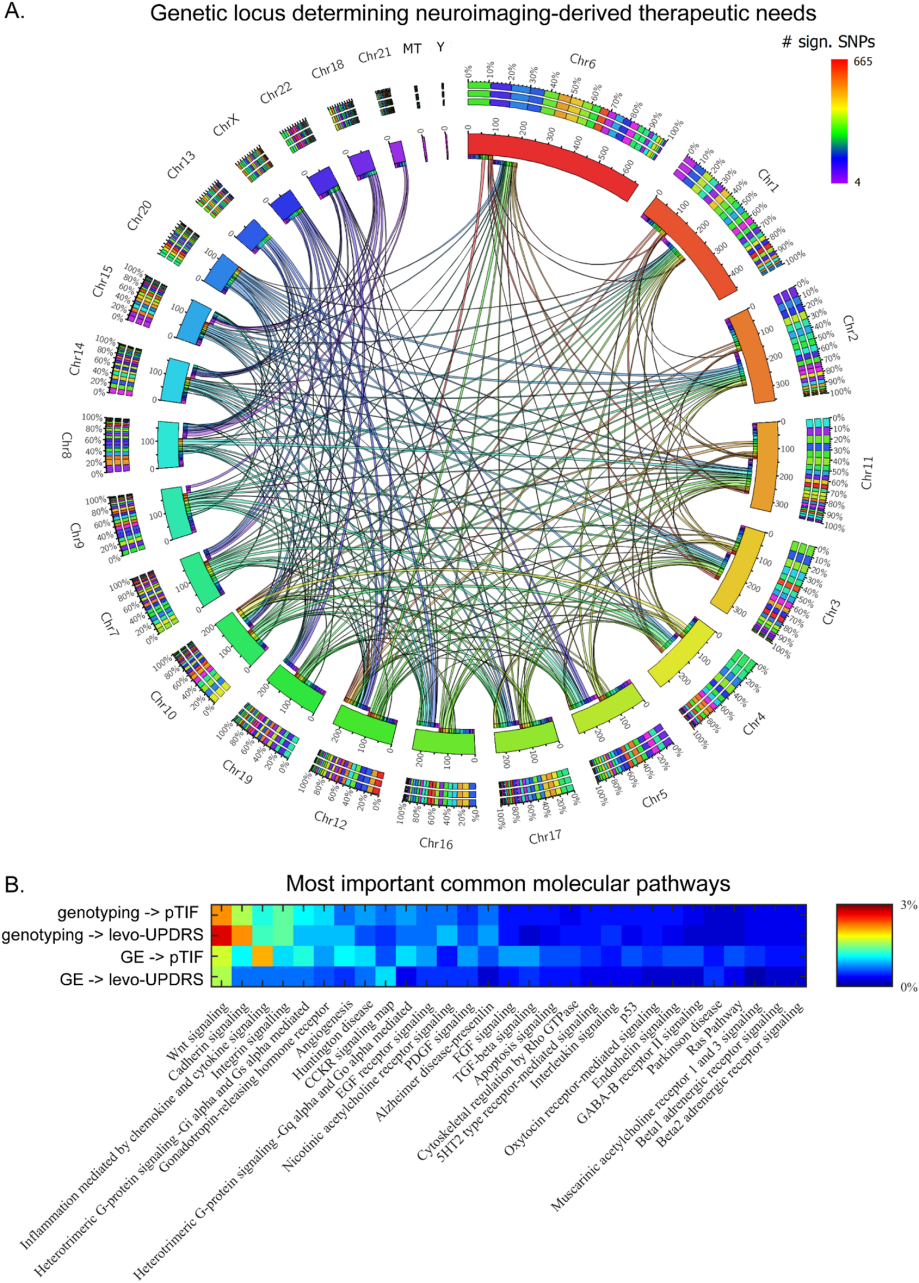
Genetic locus and molecular pathways determining neuroimaging-derived therapeutic needs in PD. (**A**) The circular plot shows the number of identified significant SNPs in each chromosome. The chromosome-chromosome links represent statistical similarity (correlation patterns) in modulation of the pTIF elements. (**B**) Top molecular pathways associated with the identified SNPs and transcripts predicting the pTIF elements and the clinical outcomes. Notice the high overlapping between the genomic and transcriptomic-based molecular predictors (see Table S2 for all the identified pathways and abbreviations used).

Next, we proceeded to identify the most statistically relevant genetic variants contributing to the pTIF prediction (see bootstrapping analysis in “Material and methods”, “Multivariate statistical analyses”). Of the 76,247 SNPs, only 5207 SNPs (associated with 4029 unique genes) significantly contributed to the covariance with the pTIF (Fig. 4A). The Protein ANalysis THrough Evolutionary Relationships (PANTHER) classification system (Mi et al., 2013) was subsequently used to identify the associated molecular pathways (see “Material and methods”, *Genetic data*). Notably, this analysis revealed 123 significant molecular pathways (Fig. 4B; Table S2) that were highly sensitive for the detection of biological processes commonly associated with neuropathological and motor deterioration mechanisms in PD. Among these, we noticed the presence of several signaling pathways: Wnt, serotonin, histamine, acetylcholine, oxytocin, thyrotropin, adrenaline and glutamate (Fig. 4B; Table S2). Other functional pathways relevant for PD included angiogenesis (linked to the formation of new blood vessels), fibroblast growth factor (FGF; among multiple functions, FGF has been shown to facilitate the formation of functional dopaminergic neurons^17^) and gonadotropin-releasing hormone receptor (GnRH; recently reported as a modulator of dopaminergic cell activity^18^). These results highlight the importance of other molecular mechanisms distinct from the classical dopaminergic system and are more clearly related to PD physiopathology and potential treatment response.

Complementarily, we also noticed (Fig. 4B; Table S2) the presence of PD and dopaminergic receptor signaling pathways coexisting with the presence of AD and Huntington’s disease pathways (e.g., presenilin- and actin-related molecular pathways, respectively). This specific finding supports the importance of studying common pathogenetic mechanisms shared by multiple overlapping neurodegenerative conditions^19^. Finally, we repeated all the analyses while adjusting for potential confounding effects due to the intake of antidiabetics, anti-inflammatory and statins drugs, which may modify PD risk and associated biomarkers^20–23^. All the results were highly similar to the described above. These included strong genomic and transcriptomic predictive associations with the imaging-derived treatment needs and observed Levodopa effects, and almost identical sets of relevant molecular pathways (Fig. S5).

### Common genetic determinants of estimated treatment needs and observed levodopa effects

Next, we aimed to explore the practical applicability of the identified molecular determinants of imaging-estimated therapeutic needs (Fig. 2, Aim 2). For this, we assumed that if the identified pTIF-predictive genetic components are accurately predictive of real personalized treatment requirements, then the individual clinical response to a specific clinical therapy (levodopa) should be significantly explained by similar molecular mechanisms. With this aim, we proceeded to characterize the causal relationship between the genetic data and longitudinal clinical effects in PD subjects under levodopa medication. For consistency, we used the same data-driven multivariate cross-correlation analysis as for the imaging-derived features and the genetic variants (i.e., SVD with randomized permutations).

Longitudinal levodopa-induced clinical effects (levo-UPDRS) were estimated for 216 PD patients using four main subscores and the total score from the MDS-UPDRS motor part (see Fig. 2, Aim 2, and “Material and methods”). Several potential confounders in treatment response assessment (measured as longitudinal slopes in UPDRS) were included as covariates (i.e., age, sex, educational level, handedness, baseline MDS-UPDRS score and LEDD; see “Material and methods”, *Clinical evaluations and treatment effects*). Similarly, for the imaging-derived data, we observed (Fig. 3D, E) that the first significant PC of the genomic information can explain up to 53.52% of the population variance in levo-UPDRS (*P* = 0.001, FWE-corrected; cross-validated added explained covariance: 21%). In addition, a bootstrapping analysis (see “Material and methods”, “Multivariate statistical analyses”) showed that all considered levo-UPDRS features were significantly predicted by the genetic data (i.e., confidence intervals did not contain zero, Fig. S3). Among the clinical outcome features, the total UPDRS score and the bradykinesia subscore were the most accurately predicted (Fig. 3F).

Once this expected causal genetic link with levodopa-induced clinical changes was confirmed^12^, we proceeded to clarify whether similar predictive molecular mechanisms/pathways were common to the imaging-derived treatment needs and the observed clinical effects. For each case, the obtained SVD loadings (or weights) were interrogated to detect which SNPs (and associated genes) in the original high-dimensional space (i.e., 76,247 SNPs) contributed the most to maximize the prediction of pTIF or levo-UPDRS (see bootstrapping analysis in “Material and methods”, “Multivariate statistical analyses”). From the 76,247 SNPs, 4053 SNPs (associated with 3168 unique genes) contributed significantly to the prediction of levo-UPDRS. Among these genes, 1028 (i.e., 33%) were common with those identified as significant predictors of imaging-derived therapeutic needs (previous subsection). In addition, we found several genes whose mutations have been previously identified via GWAS as relevant PD risks (e.g., SNCA, LRRK2, GBA and VPS13C)^24^. Gene ontology analysis using PANTHER (Mi et al., 2013) identified 115 unique functional pathways related to levo-UPDRS prediction by genotyping (Fig. 4B, Table S2). Notably, 105 (i.e. 91%) of these pathways were common to those predicting imaging-derived personalized therapeutic needs (Fig. 4B, Table S2).

### Blood gene expression predictors of estimated treatment needs and clinical outcomes

The genome contains the basic code for cell activity, but multiple mechanisms (e.g., epigenetic effects) may cause specific genes/pathways to be deactivated^25^. Finally, to filter out such cases from our previous findings, we aimed to detect whether the identified genome-based pathways (associated with individual treatment needs and treatment response in PD) were still active at the patients’ baseline evaluation. For this, blood GE was analyzed in terms of the capacity to predict both the neuroimaging-based fingerprints (N = 294) and the observed levodopa-induced clinical effects (N = 216). For statistical/methodological consistency, we used the same multivariate cross-correlation analysis, investigating the covariance between the baseline GE (34,038 transcripts) and pTIF or levo-UPDRS.

**Figure 5:**
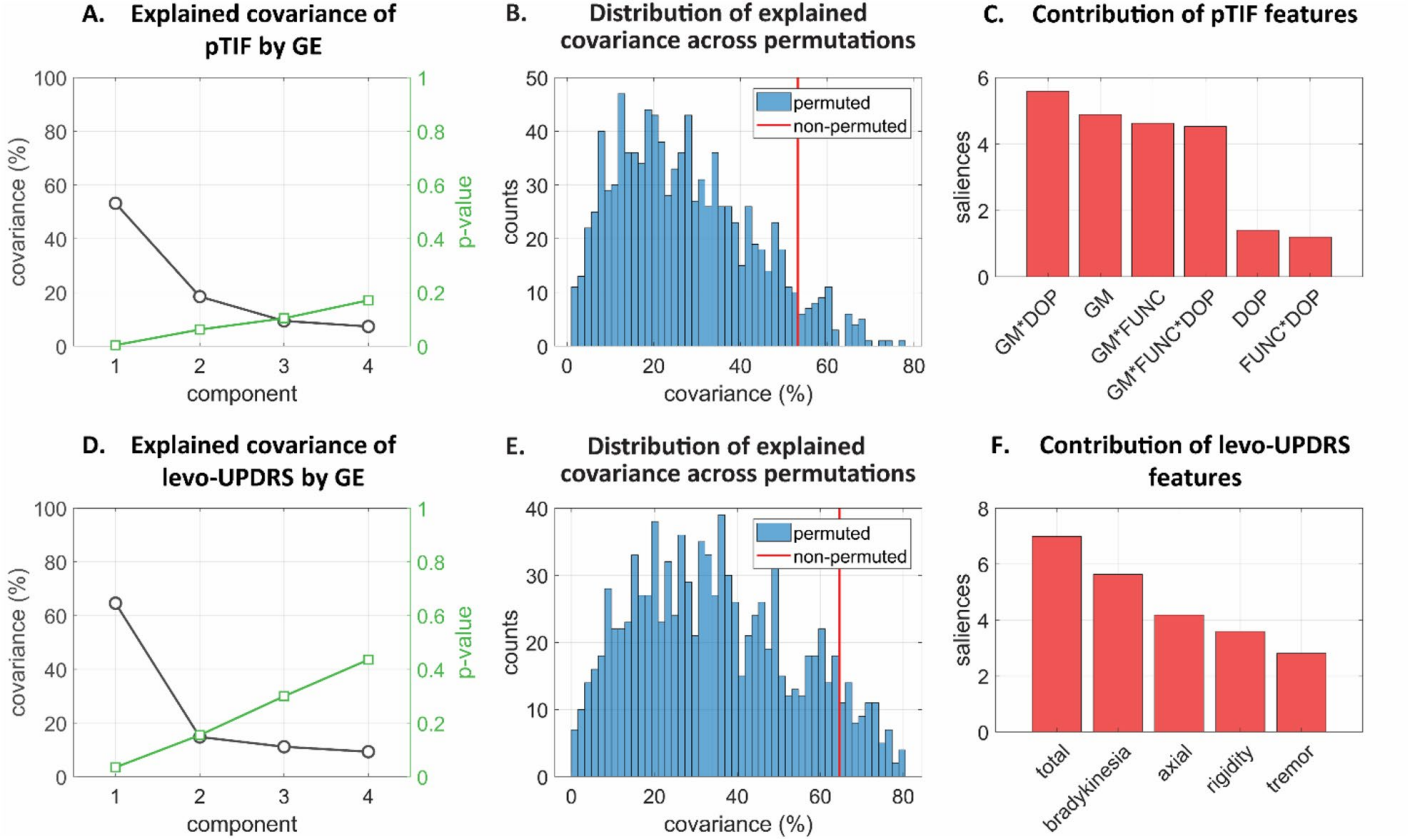
Multivariate cross-correlation results between GE, imaging-derived therapeutic needs and levodopa-induced clinical effects. (**A**) Explained covariance of pTIF by GE for the obtained principal components. (**B**) Distribution of explained covariance across randomized permutations for PC1. (**C**) Contribution of pTIF features in PC1. (**D**) Explained covariance of levo-UPDRS by GE for the principal components. (**E**) Distribution of explained covariance across permutations for PC1. (**F**) Contribution of levo-UPDRS features in PC1.

In line with our previous genomic-based results, we observed that baseline GE strongly predicted the pTIF variables, with a common explained variance of 53.21% (*P* = 0.001, FWE-corrected; cross-validated added explained covariance: 26.07%; Fig. 5B). A bootstrapping analysis revealed that all the pTIF features were significantly predicted (Fig. S2C), with features based on GM and the combination of imaging modalities being the most predictable, followed by single imaging modalities (Fig. 5C). In addition, the first GE principal component explained up to 64.61% of the population variance in levo-UPDRS (*P* = 0.002, FWE-corrected; cross-validated added explained covariance: 29.89%; Fig. 5D, E). Among the clinical outcome variables, the most comprehensive score was again the most important to covariance contribution, followed by bradykinesia and axial main subscores (Fig. 5F).

From the 34,038 gene transcripts, we identified 5944 and 3071 related genes that contributed significantly to the covariance with pTIF and levo-UPDRS, respectively. Among these genes, 41 were common to clinical outcomes and imaging-derived features. Next, the PANTHER classification system was used to identify the significant gene-associated molecular pathways. We observed a high overlap between the most important GE-based molecular pathways explaining pTIF and clinical outcomes (82% of the gene pathways contributed to levo-UPDRS covariance). Importantly, these GE-based pathways also highly overlapped (90% and 86% for pTIF and levo-UPDRS, respectively) with those based on genotypic information (Fig. 4B).

The finding of these common pathways across the main four experiments (genotyping-pTIF, genotyping-clinical, GE-pTIF and GE-clinical) evidenced the consistent gene-mediated relationship between the brain’s multifactorial reorganization and the clinical response to treatments in PD. Furthermore, the fact that the analyzed genetic variants and transcripts can similarly predict both imaging-derived therapeutic needs (i.e., pTIF variables) and observed treatment-induced clinical effects supports the crucial need to extend the genome’s further interrogation for identifying effective individually tailored treatments in PD and related disorders.

## Discussion

In this study, we defined an integrative analytic perspective to investigate the genetic and multifactorial brain basis of treatment needs and responsiveness in PD. To assess individual treatment needs, we used a novel tool consisting of imaging-based brain fingerprinting, which focuses on characterizing the brain’s complex reorganization and potential response to different therapeutic conditions^7^. Next, we evaluated the portability of the identified treatment needs and their causal genetic determinants by testing whether similar molecular mechanisms modulated clinical outcomes to levodopa. Importantly, an advanced multivariate statistical technique (bootstrapped SVD with permutation tests) allowed us to concurrently analyze several imaging features or clinical variables as interrelated genome-dependent factors in the presence of potentially pleiotropic and correlated genes. Our results evidenced a strong molecular-based overlap between neuroimaging-estimated multifactorial therapeutic needs and clinical response to levodopa. These results highlight the critical importance of simultaneously interrogating genomic, transcriptomic and neuroimaging data for identifying effective individually tailored treatments in PD, as well as for extending the pathogenetic understanding of this heterogeneous disease.

In this decade, the identification of distinctive disease subtypes in PD has become a primary research topic, mainly motivated by the purpose of selecting subtype-specific treatments and maximizing therapeutic effects^26^. For this purpose, clinical, imaging, genetic mutation, peripheral biomarker and neuropathological data have been considered, often using a unifactorial perspective^27^. A traditional procedure is to cluster patients according to their similarity at a given time point/age or based on a unique type of information (e.g., clinical, imaging or genetic). Novel attempts are moving towards including several biomarkers and time points for patient clustering via advanced machine learning techniques^28^. We aimed to extend such analyses in multiple directions. First, by considering that the patients may need different treatments depending not only on their brain's multifactorial alterations (e.g., dopaminergic neuronal loss, functional dysregulation, atrophy) but also on how the different biological factors interact and how they could respond (at the individual level) to different clinical interventions^7^. This has provided a simplified individual patient profile of the quantitative biological factor modifications needed to control disease evolution (here referred to as pTIF). Second, by decomposing this personalized profile in terms of its causal genetic determinants, we went beyond an estimate of the patient-specific therapeutic needs to obtain a molecular roadmap of the genetic predisposition underlying interindividual treatment differences in the `same’ disease.

Our analysis showed that the genomic data can significantly explain (*P* < 0.01, FWE-corrected; Fig. 3A, D) the population variability in the imaging-derived pTIF and levo-UPDRS, respectively. Similar results were obtained when using blood GE as a predictor (Fig. 5), confirming the active role of the identified genomic-based molecular pathways at the time of patient evaluation (Figs. 3, 5). In both cases (i.e., using DNA or RNA), the relatively lower prediction accuracy obtained for clinical evaluations may be attributed to the superior sensitivity of the defined neuroimaging metrics compared to the UPDRS. By definition, the multimodal imaging fingerprint comprised a higher number of features (351 regions for each imaging modality) than the UPDRS motor part. Clinical outcome measurements such as UPDRS scores also suffer from a substantial amount of within-subject variability^29^, which could be due to measurement error and short-term drug effects. Lastly, we confirmed that clinical outcomes were driven by levodopa-like medication effect. In fact, we replicated our multivariate analysis with absolute levodopa response (aLR) which assessed the difference between UPDRS motor scores after vs before drug administration^30^. The first components explained about 46% and 54% of covariance with genotyping and GE, respectively (*P* = 0.001 and *P* = 0.004, FWE-corrected; Fig. S3 and S2E, F).

Similarly to what has been done in Alzheimer’s disease^31^, this study used robust multivariate statistical analyses for identifying large clusters of functionally related genetic determinants. Traditionally, GWAS in PD have identified single risk-related SNPs or loci, focusing on a unique phenotypic or clinical trait/aspect at a particular time and, consequently, have been less practical for discovering clusters of functionally related genes and pathways^13^. By concurrently analyzing several imaging-derived or clinical domains, our multivariate analysis searched for large clusters of functionally related SNPs in the presence of potentially pleiotropic and correlated genes. The applied SVD method (and its associated permutation test) focused on identifying the specific set of gene variants maximally related to the multivariate treatment needs or the response scores while controlling for the covariates. Remarkably, most of the identified gene pathways are involved in signal transduction. This is particularly relevant considering the growing interest in signaling factors to explain PD mechanisms^32^. While some of these pathways (e.g., dopamine receptor signaling) are expected to be strong predictors of PD disease progression and treatment response, other pathways, such as those related to AD (e.g., presenilin pathway), suggest the need to further consider pleiotropy^33^. As one gene usually affects more than one phenotype, it is reasonable to think that biological markers of PD progression and heterogeneity could be affected by genetic pathways that are clearly distinct from dopaminergic and other pathways less commonly mentioned in PD research.

Our study also has a number of limitations. Potential pleiotropy is a limiting factor to consider, in particular, the exclusive use of brain-focused imaging and motor symptoms. To obtain the most reliable results, we chose to select specific structural, functional and molecular brain imaging features, as they are well known to be altered based on PD progression^34^. However, PD-related genes can also modulate nonbrain phenotypes. For instance, new evidence suggests the existence of two main PD types: brain-first and body-first^35^. In the latter case, brain imaging would not necessarily be sensitive enough to detect early PD alterations and associated therapeutic needs. In addition, the fact that we used a structural connectome from healthy participants data may also decrease our ability to capture multimodal brain reorganization in disease, which may subsequently bias the predicted treatment needs. Similarly, we chose the UPDRS motor part for its well-established relationship and reliability in PD evaluation^30,36^. However, the selection of only motor outcomes may have prevented us from detecting other causal genes associated with the impact of treatment on cognitive decline. Due to the lack of available datasets with equivalent multimodal data types to PPMI, cross-validation in an independent PD population was not performed. This represents an indispensable future step to corroborate our findings. Finally, levodopa and equivalent drugs are only one of the several practiced treatments for PD^37,38^. Future work may include other types of interventions, such as deep brain stimulation and stem cell therapy, to find the best individually tailored therapy according to genomic data and imaging-derived therapeutic needs. In summary, our multivariate data-driven analyses allowed the genome-based decomposition of brain imaging-derived therapeutic needs and motor clinical outcomes in PD. We also detected novel genes and related molecular pathways with the impact of guiding the development of new drug agents. Future studies should include additional information, such as whole-body imaging, cognitive treatment outcomes and other treatments, with the ultimate goal of accelerating the implementation of precision medicine in PD.

## Material and methods

### Data description and processing

Data used in the preparation of this article were obtained from the Parkinson’s Progression Markers Initiative (PPMI) database in July 2019 (www.ppmi-info.org/access-dataspecimens/download-data). For up-to-date information on the study, visit ppmi-info.org. PPMI contained a large dataset of subjects from multiple medical centers around the world. At the first visit, all subject received diagnostic related to PD disease and demographics were collected. Next, they underwent several visits where multimodal imaging, genetics and clinical data were acquired.

PPMI subjects provided written, informed consent to participate and all PPMI study was conducted in accordance with the Declaration of Helsinki and the Good Clinical Practice (GCP) guidelines after approval of the local ethics committees of the participating sites^39^. Authors obtained the permission to use PPMI data for this study.

### Study participants

We used two datasets. The first dataset contained subjects with longitudinal imaging data, while the second dataset contained subjects with longitudinal clinical outcomes. For the first dataset, 1000 participants were included with at least one imaging dataset. The imaging-derived individual therapeutic needs estimation (see “Material and methods”, “Imaging-derived individual therapeutic needs estimation”) was estimated for 362 subjects with at least three imaging modalities and three time points that survived the quality control. From this sample, we selected 294 subjects diagnosed with PD who had baseline genetic data, clinical evaluation, medication data and demographics (see flow chart, Fig. S1). The second dataset was based on 216 subjects diagnosed with PD who had at least 3 UPDRS ON-dose evaluations, baseline genetic data, medication data and demographics. Note that 181 subjects are common between the first and second datasets. Demographics (sex, race, handedness, age, education), diagnostic groups, baseline UPDRS scores and medication data are summarized in Table S1. None of the subjects in the two final datasets were from the PPMI cohort that contains people with PD and pathogenic genetic variant(s) in LRRK2, GBA and rare genetic variants.

### Genetic data

#### Genome-wide genotyping

Single nucleotide polymorphisms (SNPs) genotyping was performed using Illumina NeuroX array on 619 whole-blood extracted DNA samples collected according to the PPMI Research Biomarkers Laboratory Manual. The NeuroX array is an Illumina Infinium iSelect HD Custom Genotyping array containing 267,607 Illumina standard content exonic variants and an additional 24,706 custom variants designed for neurological disease studies. Of the custom variants, approximately 12,000 are designed to study Parkinson’s disease and are applicable to both large population studies of risk factors and to investigations of familial disease and known mutations. Quality control of sample handling was determined by comparing the subject’s sex reported by Coriell Institute for Medical Research with the genotypic sex estimated from X chromosome heterogeneity. From these 267,607 and 24,706 variants, we selected 76,247 variants that survive to quality check (i.e. consistency between replicates) and have a non-null variability between subjects. We obtained corresponding genes and regulatory regions based on their chromosome position and reference/alternative bases with Ensembl database (GRCh37 assembly). Only strictly overlapped genes were selected (0 bp distance upstream or downstream). For SNPs overlapping regulatory regions, we converted their chromosome position with NCBI Genome Remapping service to GRCh38 (https://www.ncbi.nlm.nih.gov/genome/tools/remap) and got the linked genes with the EpiRegio database^40^. However, some of the SNPs were not linked to any gene because they were not overlapping any regulatory region (418 and 339 SNPs among significant SNPs from pTIF and levo-UPDRS analyses, respectively) or their overlapped regulatory regions were not linked to any gene (78 and 64 SNPs among significant SNPs from pTIF and levo-UPDRS analyses, respectively).

#### Gene expression

Whole-transcriptome RNA-seq was performed from 1 ug aliquots of RNA isolated from PaxGene tubes. Data was sequenced at Hudson Alpha's Genomic Services Lab on an Illumina NovaSeq6000. All samples went through rRNA + globin reduction, followed by directional cDNA synthesis using the NEB kit. Following second‐strand synthesis, the samples were prepped using the NEB/Kapa (NEBKAP) based library prep. BCL’s were converted to using bcltofastq v1.8.4, and FASTQ’s were merged and aligned to Full Reference Genome Sequence hs37d5 (GRCh37 assembly) by STAR (v2.4K) on GENCODE v19.

As an interim analysis, quality control steps were conducted, and two types of primary results files were created consisting of abundance estimates (Transcripts per Million, TPM) via Salmon. TPMs was selected as a normalized abundance estimate for querying 34,038 genes that have a non-null variability between subjects.

#### Gene classification

We used PANTHER (Protein ANalysis THrough Evolutionary Relationships) classification system to classify genes in pathways that specify the main molecule implicated^41^.

### Clinical evaluations and treatment effects

For each participant, the third motor part of the Movement Disorder Society-Unified Parkinson's Disease Rating Scale (MDS-UPDRS), a revision of the Unified Parkinson's Disease Rating Scale (UPDRS), was evaluated at each visit. In order to make our results meaningful to clinicians, we grouped the 33 motor part scores to four main subscores (tremor, bradykinesia, rigidity and axial), in addition to the total UPDRS third motor part score, as described in a previous study^42^.

Next, in order to quantify treatment-induced clinical outcomes changes over time, a linear polynomial across the time points of each subject was fitted, taking the slope term/parameter as a direct measure of treatment effects. Importantly, in order to consider confounders in treatment outcome assessment, in our singular value decomposition (SVD) multivariate statistical analysis, we adjusted for the patients’ age, sex, education level, handedness, baseline MDS-UPDRS motor score and Levodopa Equivalent Daily Dose (LEDD). LEDD was obtained from the whole concurrent medication of each patient. Using keyword and matching between medication names and its indications, we extracted dose, unit and frequency for each PD medication. Next, we computed LEDD according to previous studies^43–48^ and information available from FDA or its international counterparts. All data were checked for consistency between and within subjects.

### Multimodal imaging data acquisition and preprocessing

We selected 1000 subjects which have at least one acquisition of structural MRI (T1), resting state fMRI and/or SPECT imaging data (see flow chart, Fig S1).

#### Structural MRI

Brain structural T1-weighted 3D images were acquired for 890 subjects. For a detailed description of acquisition details, see https://www.ppmi-info.org/wp-content/uploads/2017/06/PPMI-MRI-Operations-Manual-V7.pdf. Similarly to previous study^49^, images underwent non-uniformity correction using the N3 algorithm. Next, they were segmented into grey matter, white matter and cerebrospinal fluid (CSF) probabilistic maps, using SPM12 (www.fil.ion.ucl.ac.uk/spm). Grey matter density segmentations were standardized to MNI space^50^ using the DARTEL tool^51^. Each map was modulated in order to preserve the total amount of signal/tissue. Visual quality control for aliasing, registration and brain abnormalities removed 19 images resulting in 16 excluded subjects. Mean grey matter density (GM) values were calculated for 351 regions covering all the brain’s grey matter^52,53^.

#### Resting fMRI

Resting-state functional images were obtained for 189 subjects (354 images) using an echo-planar imaging sequence on a 3.0-T Siemens MRI scanner. Acquisition parameters were: 210 time points, repetition time (TR) = 2400 ms, echo time (TE) = 25 ms, flip angle = 80°, number of slices = 40, spatial resolution = 3.3 × 3.3 × 3.3 mm^3^ and in plane matrix = 68 × 66. Preprocessing steps included: (1) motion correction, (2) slice timing correction, (3) spatial normalization to MNI space^50^ using the registration parameters obtained for the structural T1 image with the nearest acquisition date, and (4) signal filtering to keep only low frequency fluctuations (0.01–0.08 Hz)^54^. Visual quality control removed 3 images and excluded 1 subject. We carefully identified subjects that could be on medication (n = 140 subjects). In order to have regional quantitative indicators of the brain’s functional integrity, fractional amplitude of low-frequency fluctuation (fALFF)^55^ was calculated for each of the 351 considered brain regions^52,53^.

#### DatSCAN SPECT

Preprocessed DatSCAN SPECT images were obtained for 748 subjects from the PPMI database. The preprocessing included normalization and registration to the MNI space, as described in the whitepapers in the study information^56^. All the scans went through visual inspection and in case of misregistration, we further, registered the subject to the average linear template of the healthy subjects. The healthy template was created using averaging and linear registration to the average of SPECT scans which was then registered to the MNI template. The combination of registrations was then used to realign the mis-registered scans. All subjects were again visually inspected for quality control, and 88 images and 4 subjects were excluded. Finally, for each subject and time point, average dopaminergic integrity values were calculated for the 351 considered cortical and subcortical brain regions^52,53^.

#### Whole-brain connectivity estimation

The connectivity matrix was constructed using DSI Studio, based on a deterministic fiber tracking algorithm that leverages information in the spin distribution function^57^. A high angular and spatial resolution diffusion-weighted imaging template was previously constructed^58^ from a total of 1065 healthy subjects (575 female, average age: 28.74) from the Human Connectome Project. A multishell diffusion scheme was used, with b-values 990, 1985 and 2980 s/mm^2^. The number of diffusion sampling directions were 90, 90, and 90, respectively. The in-plane resolution was 1.25 mm. The slice thickness was 1.25 mm. The diffusion data were reconstructed in the MNI space using q-space diffeomorphic reconstruction^58^ to obtain the spin distribution function^59^. A diffusion sampling length ratio of 2.5 was used, and the output resolution was 1 mm.

### Imaging-derived individual therapeutic needs estimation

For each study participant with longitudinal imaging data (N = 362), we aimed to estimate the individual multifactorial brain reformations required for stopping her/his brain deterioration over a one-year period (i.e., keeping the subject’s brain properties in a stationary state). For this, we used the concept of *personalized* therapeutic fingerprints (pTIFs)^7^. The pTIF assumes that the patients may need different treatments, not only depending on their brain's unifactorial alterations (e.g., dopamine alteration or not, functional dysregulation or not, atrophy or not) but also on their individual multifactorial brain dynamics: how the different biological factors interact and how they could respond (at the individual level) to potential clinical perturbations^7^. Based on the spatiotemporal analysis of multimodal imaging data (i.e., T1-MRI, fMRI, SPECT), pTIF values are a set of multivariate metrics that reflect the biological reformation required to stop the pathologic progression or revert the condition to normality. Note that when using three imaging modalities (T1-MRI, fMRI and dopamine SPECT), the number of all possible single-target or combinatorial interventions (up to a maximum of 3 biological factors/modalities) is 7. As a result, we obtained 7 global pTIF values corresponding to the required whole-brain reformations for targeting gray matter density (GM), functional activity at rest (FUNC, quantified as fALFF), dopaminergic integrity (DOP), GM-FUNC, GM-DOP, FUNC-DOP and GM-FUNC-DOP. Of note, the global pTIF was estimated with the *Neuroinformatics for Personalized Medicine* toolbox (*NeuroPM-box*^60^, available at www.neuropm-lab.com/software).

### Multivariate statistical analyses

To evaluate the covariate-adjusted association of the high-dimensional genetic data (genotyping and GE) with the clinical-effects data or the imaging-derived therapeutic needs (see the pTIF concept above), we used a data-driven multivariate cross-correlation analysis in combination with a randomized permutation test^61,62^. Specifically, we applied SVD^14^, a technique that generalizes both principal component analysis (PCA)^15^ and partial least squares (PLS)^16^, to the case of genetic and clinical/imaging features. The SVD seeks to express the cross-correlation structure between any two sets of variables by a small number of pairs of “principal components” (PCs), with each PC associated with weights or loadings that vary across features^14^.

For pTIF and clinical outcome variables, covariates were age, sex, education level, handedness, baseline MDS-UPDRS motor score and LEDD. There was no covariate for genotyping and gene expression. We removed covariates (*cov*) from variables by doing robust regression, estimating each feature in variable with covariates, then taking the sum of intercept and residuals as covariate adjusted variable. We also run analyses without covariates removal and got similar results. Normality assumptions were reached by applying Box-Cox transformation and z-score rescaling for both covariates and variables. In addition, for genotyping analyses, we computed the ranks of the pTIF and clinical outcome variables in order to deal with the persistent discrete distribution of genotyping data. In supplementary analyses, we also adjusted for potential confounding effects due to antidiabetics, ant-inflammatory and statins drugs intake, which may modify PD risk and associated biomarkers^20–23^. Dummy variables reflecting the individual use of these drugs were added to the covariables and removed before each SVD calculation, as described before.

Subsequently, while accounting for covariates, this method allowed us to evaluate the statistical association of the individual genetic features (i.e., predictor variables) with (1) imaging-inferred treatment needs and (2) treatment-induced clinical effects. For each of these two scenarios (i.e., genetics vs imaging features and genetics vs clinical effects) a permutation with 1000 iterations was executed to determine the statistical significance of each principal component after SVD^16^. Cross-validated added covariance was computed as the difference between the original (non-permuted) explained variance and the mean of the randomly permuted explained variances. Bootstrapping with 1000 iterations was used to assess the importance and significance of each feature by reporting their bootstrapping ratio and CI, respectively^16^. The ratio was calculated as the original weight of a feature divided by its standard error across all bootstrapping iterations. Features with CIs that did not contain zero were considered statistically significant. This analysis was designed for each of the two described scenarios to investigate whether genetic features could predict imaging features and clinical effects.

## Supplementary Information

Supplementary Information.

## Data Availability

Data used for this paper were obtained from the Parkinson’s Progression Markers Initiative database (PPMI, www.ppmi-info.org/data). PPMI provides full, open access to all investigators in the scientific community.

## Code availability

Analytical methods for pTIF estimation are available for academic researchers as part of the open-access cross-platform user-friendly *Neuroinformatics for Personalized Medicine* toolbox (*NeuroPM-box*, freely available at www.neuropm-lab.com/software^60^).
