# Supplementary material for: Multivariate genomic and transcriptomic determinants of imaging-derived personalized therapeutic needs in Parkinson’s disease
Source: Sci Rep. 2022 Mar 31;12:5483. doi: 10.1038/s41598-022-09506-0 (PMC8971452; doi:10.1038/s41598-022-09506-0)
Supplement: Supplementary file 1 — Supplementary Information. [file 41598_2022_9506_MOESM1_ESM.docx]

**Supplementary Information**


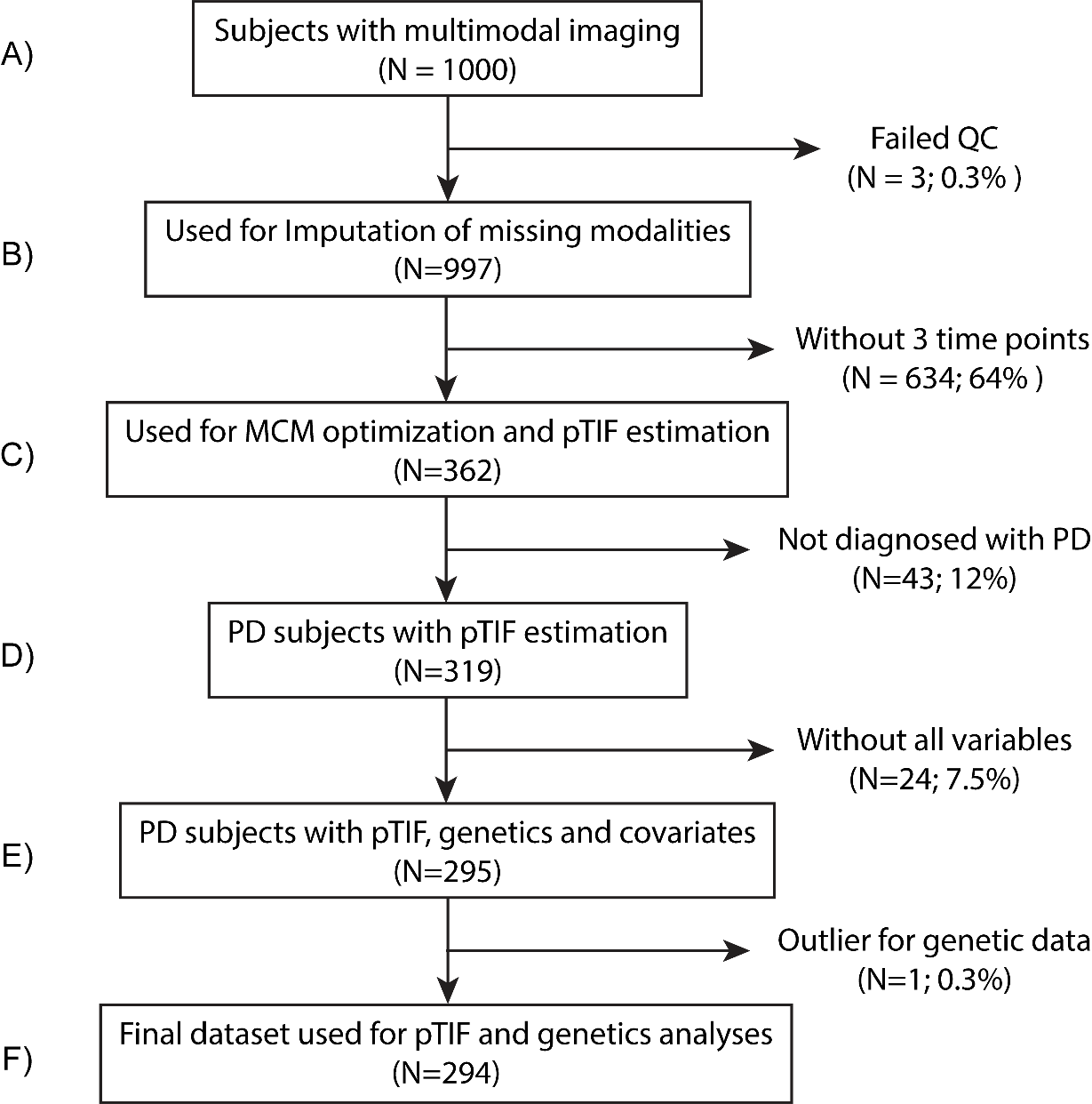


Figure S1. Flowchart of the main steps followed for participant selection and analysis. A) 1000 subjects from PPMI, with at least one imaging modality, were included. Next, image preprocessing followed by quality control excluded 3 subjects. B) 997 remaining subjects were used for imputation of imaging modalities C) In order to capture dynamic changes at the individual level, only subjects with at least 3 imaging time points (N=362) were kept for the subsequent next analysis. The MCM optimization algorithm and pTIF estimation were applied for these 362 participants. D) Among these subjects, 319 were diagnosed with PD. E) 295 subjects had data for each variable (genetic and clinical data). F) One outlier for genetics data was removed to obtain the final dataset with 294 PD subjects. For further details, see *Materials and Methods* section.


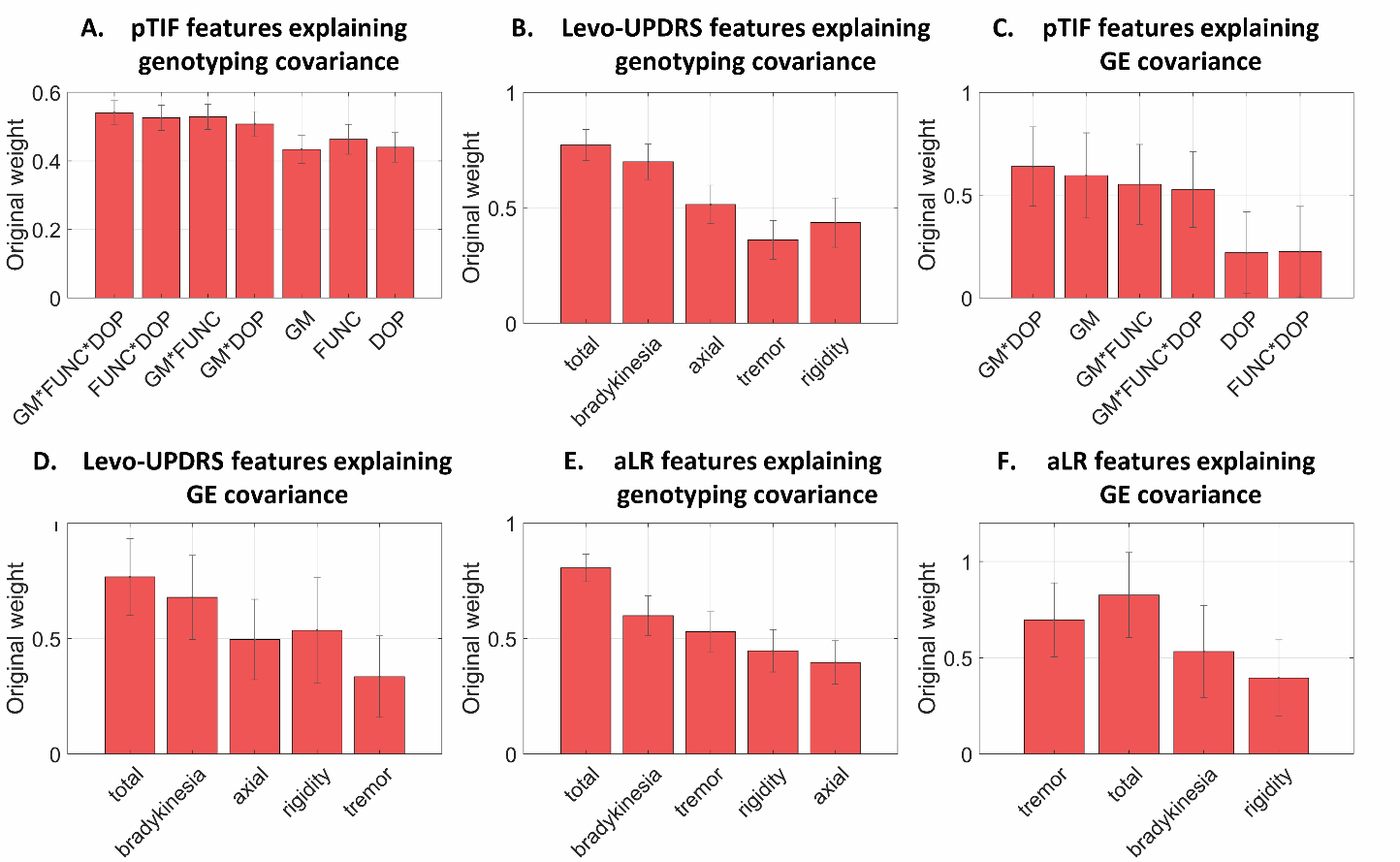


Figure S2. Mean bootstrap ratio weight with 95% confidence intervals computed. A) Global pTIF features that explain genotyping covariance. B) Levo-UPDRS features that explain genotyping covariance. C) pTIF features that explain GE covariance. D) Levo-UPDRS features that explain GE covariance. E) aLR features that explain genotyping covariance. F) aLR features that explain GE covariance.


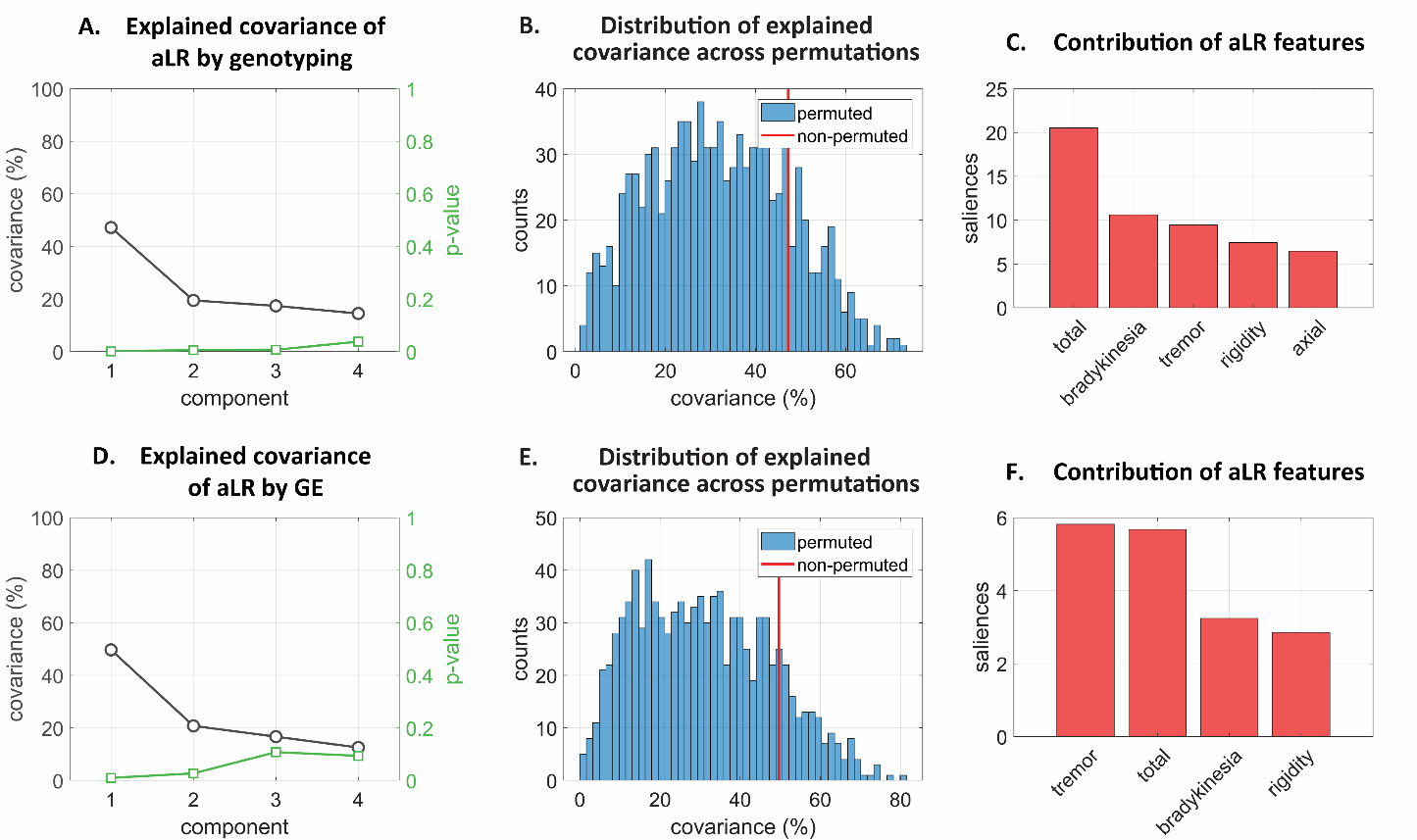
Figure S3. Multivariate cross-correlation results between genotyping and aLR. A) Explained covariance of aLR by genotyping for each principal component. B) Distribution of explained covariance across permutations for the first significant principal component (PC1). C) Contribution of aLR features in PC1. D) Explained covariance of aLR by GE for the obtained principal components. E) Distribution of explained covariance across permutations for PC1. F) Contribution of aLR features in PC1.


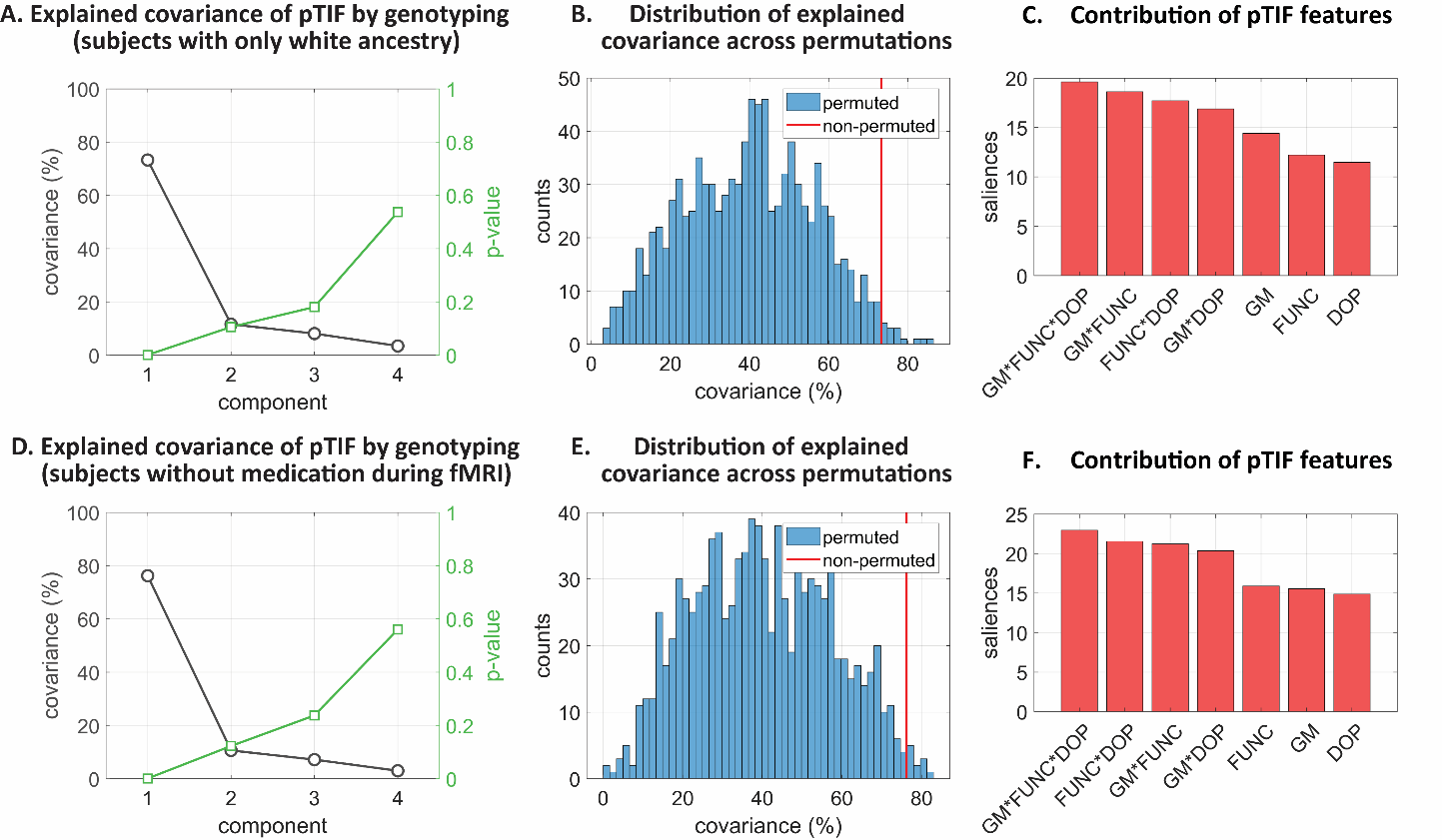


Figure S4. Cross-correlation results for patients from white-race or on off-medication fMRI. A) Explained covariance of pTIF by genotyping for each principal component in white-race. B) Distribution of explained covariance across permutations for the first significant principal component (PC1). C) Contribution of pTIF features in PC1. D) Explained covariance of pTIF by genotyping for each principal component in patients without medication during fMRI. E) Distribution of explained covariance across permutations for the first significant principal component (PC1). F) Contribution of pTIF features in PC1. Notice the strong similarity/consistency with the results obtained for the whole-population (Fig. 3).


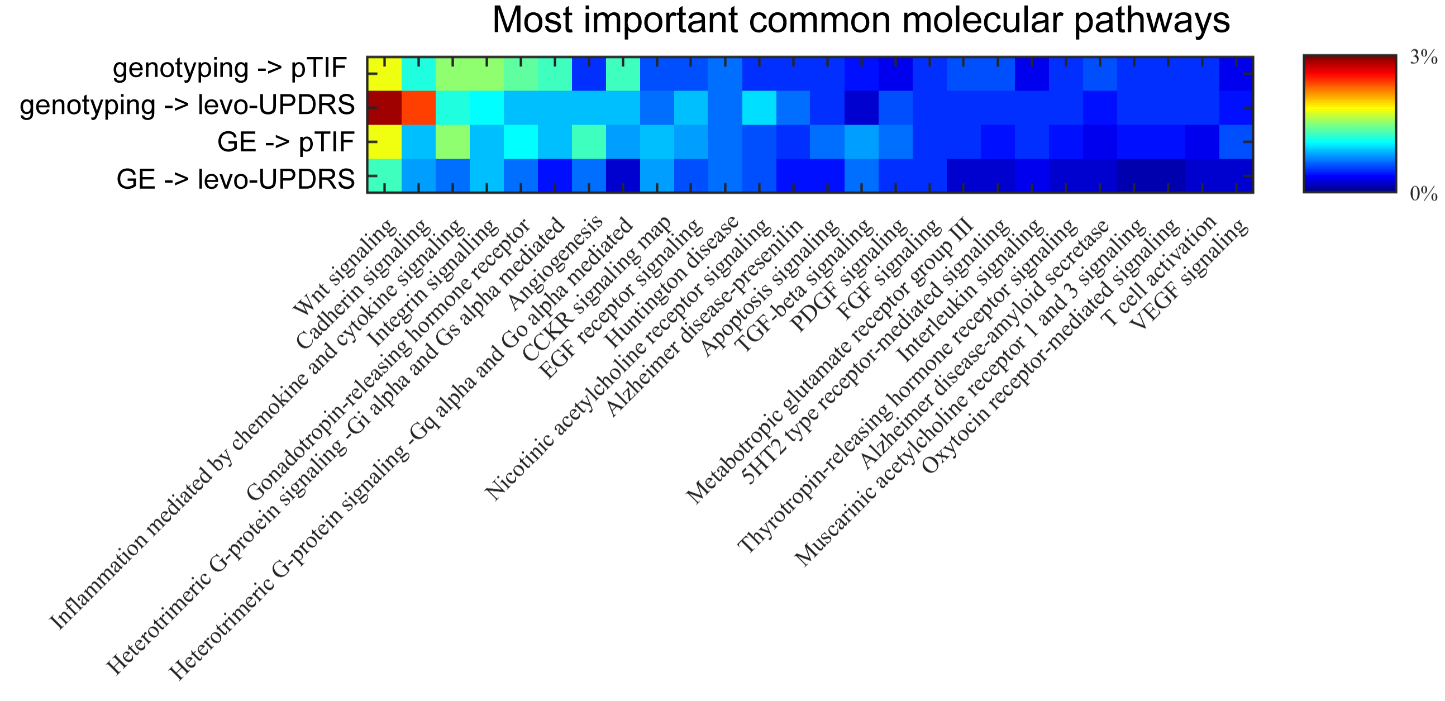


Figure S5. Top molecular pathways determining therapeutic needs in PD after adjusting for additional drugs intake (antidiabetics, anti-inflammatory and statins). Notice the high overlapping between the genomic and transcriptomic-based molecular predictors, as well as the consistency with un-adjusted results (Fig. 4B).

Table S1. Characteristics of the included PPMI subjects.

| **variable** | **Participants with imaging data**  (n = 997) | **PD patients with pTIF estimations**  (n=294) | **PD patients with longitudinal clinical data**  (n=216) |
| --- | --- | --- | --- |
| Men | 607 (61%) | 199 (68%) | 147 (68%) |
| With only white ancestry | 914 (92%) | 283 (96%) | 201 (93%) |
| Without white ancestry | 54 (5%) | 5 (2%) | 9 (4%) |
| Right-handed | 854 (86%) | 255 (87%) | 190 (88%) |
| Baseline age  (years) | 68.3 (9.86) | 68.79 (9.96) | 68.92 (9.68) |
| Education  (years) | 15.86 (3.34) | 15.66 (2.84) | 15.7 (3.03) |
| Control | 215 (22%) | 0 | 0 |
| GenCohortPD | 88 (9%) | 0 | 0 |
| *de novo* | *12* | *0* | *0* |
| GenCohortUnaff | 107 (11%) | 0 | 0 |
| GenRegistPD | 1 (0.1%) | 0 | 0 |
| GenRegistUnaff | 3 (0.3%) | 0 | 0 |
| PD | 457 (46%) | 294 | 216 |
| *de novo* | *235* | *149* | *0* |
| Prodromal | 50 (5%) | 0 | 0 |
| SWEDD | 76 (8%) | 0 | 0 |
| Baseline UPDRS  (all parts) | 23.16 (17.89) | 32.35 (13.07) | 36.43 (16.61) |
| Baseline UPDRS  (motor part) | 13.28 (11.79) | 20.79 (8.51) | 21 (11.3) |
| Baseline LEDD | 100.91 (209.6) | 162.09 (134.82) | 176.85 (161.21) |
| Subjects taking medication such as:  statin | 259 (26%) | 78 (27%) | 67 (31%) |
| Antidiabetic | 72 (7%) | 17 (6%) | 13 (6%) |
| Anti-inflammatory | 20 (2%) | 5 (2%) | 6 (3%) |

Data are number (%) or mean value (std). De novo PD are defined as no PD medication before the first imaging data and six months later [1]. GenCohortPD: cohort containing subjects with a pathogenic genetic risk variant (LRRK2, GBA, SNCA) and diagnosed with PD; GenCohortUnaff: cohort containing subjects with a pathogenic genetic risk variant but not diagnosed with PD; GenRegistPD: registry containing subjects with a pathogenic genetic risk variant and diagnosed with PD; GenRegistUnaff: registry containing subjects with a pathogenic genetic risk variant but not diagnosed with PD.

Table S2. Common molecular pathways related to significant genotyping and gene expression, and, underlying imaging-derived personalized therapeutic needs and clinical outcomes.

| **Pathway name** | **Presence (%)** | | | |
| --- | --- | --- | --- | --- |
|  | genotyping -> pTIF | genotyping -> levo-UPDRS | GE -> pTIF | GE -> levo-UPDRS |
| Wnt signaling | 2.2 | 2.7 | 1.7 | 1.6 |
| Cadherin signaling | 1.6 | 2.2 | 1.1 | 0.7 |
| Inflammation mediated by chemokine and cytokine signaling | 1.2 | 1.3 | 2.1 | 0.7 |
| Integrin signaling | 1.4 | 1.4 | 1 | 0.7 |
| Heterotrimeric G-protein signaling -Gi alpha and Gs alpha mediated | 1.1 | 0.9 | 1.2 | 0.7 |
| Gonadotropin-releasing hormone receptor | 1 | 0.9 | 0.8 | 0.6 |
| Angiogenesis | 0.7 | 0.9 | 1.1 | 0.5 |
| Huntington disease | 0.8 | 0.6 | 1 | 0.7 |
| CCKR signaling map | 0.7 | 0.5 | 0.6 | 1 |
| Heterotrimeric G-protein signaling -Gq alpha and Go alpha mediated | 0.8 | 0.7 | 1 | 0.3 |
| EGF receptor signaling | 0.7 | 0.7 | 0.8 | 0.5 |
| Nicotinic acetylcholine receptor signaling | 0.7 | 0.9 | 0.4 | 0.5 |
| PDGF signaling | 0.5 | 0.6 | 0.8 | 0.5 |
| Alzheimer disease-presenilin | 0.7 | 0.8 | 0.6 | 0.2 |
| FGF signaling | 0.4 | 0.4 | 0.8 | 0.4 |
| TGF-beta signaling | 0.4 | 0.2 | 0.8 | 0.5 |
| Apoptosis signaling | 0.4 | 0.3 | 0.6 | 0.4 |
| Cytoskeletal regulation by Rho GTPase | 0.3 | 0.4 | 0.6 | 0.4 |
| 5HT2 type receptor-mediated signaling | 0.4 | 0.4 | 0.5 | 0.3 |
| Interleukin signaling | 0.4 | 0.5 | 0.4 | 0.3 |
| p53 | 0.4 | 0.3 | 0.6 | 0.3 |
| Oxytocin receptor-mediated signaling | 0.4 | 0.4 | 0.5 | 0.2 |
| Endothelin signaling | 0.4 | 0.3 | 0.6 | 0.2 |
| GABA-B receptor II signaling | 0.3 | 0.2 | 0.6 | 0.2 |
| Parkinson disease | 0.2 | 0.2 | 0.4 | 0.5 |
| Ras Pathway | 0.2 | 0.2 | 0.6 | 0.3 |
| Muscarinic acetylcholine receptors 1 and 3 signaling | 0.3 | 0.3 | 0.5 | 0.1 |
| Beta1 adrenergic receptor signaling | 0.3 | 0.3 | 0.4 | 0.2 |
| Beta2 adrenergic receptor signaling | 0.3 | 0.3 | 0.4 | 0.2 |
| Metabotropic glutamate receptor group III | 0.2 | 0.4 | 0.4 | 0.2 |
| Thyrotropin-releasing hormone receptor signaling | 0.3 | 0.3 | 0.4 | 0.2 |
| VEGF signaling | 0.2 | 0.3 | 0.5 | 0.2 |
| Alzheimer disease-amyloid secretase | 0.4 | 0.4 | 0.1 | 0.2 |
| Oxidative stress response | 0.3 | 0.1 | 0.1 | 0.6 |
| Toll receptor signaling | 0.3 | 0.1 | 0.4 | 0.3 |
| Transcription regulation by bZIP transcription factor | 0.2 | 0.1 | 0.4 | 0.4 |
| B cell activation | 0.2 | 0.2 | 0.4 | 0.2 |
| Dopamine receptor-mediated signaling | 0.2 | 0.1 | 0.6 | 0.1 |
| Histamine H1 receptor-mediated signaling | 0.2 | 0.3 | 0.4 | 0.1 |
| Muscarinic acetylcholine receptors 2 and 4 signaling | 0.2 | 0.3 | 0.4 | 0.1 |
| p53 feedback loops 2 | 0.2 | 0.2 | 0.4 | 0.2 |
| 5HT1 type receptor-mediated signaling | 0.2 | 0.1 | 0.4 | 0.2 |
| 5HT4 type receptor-mediated signaling | 0.2 | 0.1 | 0.4 | 0.2 |
| T cell activation | 0.2 | 0.4 | 0.1 | 0.2 |
| Blood coagulation | 0.4 | 0.3 | 0.1 | 0.1 |
| Axon guidance mediated by semaphorins | 0.2 | 0.1 | 0.2 | 0.3 |
| Metabotropic glutamate receptor group II | 0.1 | 0.2 | 0.4 | 0.1 |
| Opioid proenkephalin | 0.1 | 0.1 | 0.4 | 0.2 |
| Opioid proopiomelanocortin | 0.1 | 0.1 | 0.4 | 0.2 |
| Ubiquitin proteasome | 0.1 | 0.1 | 0.4 | 0.2 |
| Beta3 adrenergic receptor signaling | 0.1 | 0.1 | 0.4 | 0.1 |
| Enkephalin release | 0.1 | 0.1 | 0.4 | 0.1 |
| Heterotrimeric G-protein signaling -rod outer segment phototransduction | 0.1 | 0.1 | 0.4 | 0.1 |
| Interferon-gamma signaling | 0.2 | 0.1 | 0.3 | 0.1 |
| Ionotropic glutamate receptor | 0.1 | 0.3 | 0.2 | 0.1 |
| Opioid prodynorphin | 0.1 | 0.1 | 0.4 | 0.1 |
| PI3 kinase | 0.2 | 0.1 | 0.4 | 0 |
| Angiotensin II-stimulated signaling through G proteins and beta-arrestin | 0.2 | 0.1 | 0.2 | 0.2 |
| Axon guidance mediated by netrin | 0.2 | 0.2 | 0.1 | 0.2 |
| DNA replication | 0.1 | 0.1 | 0.3 | 0.2 |
| Insulin/IGF -protein kinase B signaling cascade | 0.2 | 0.3 | 0.1 | 0.1 |
| Nicotine pharmacodynamics | 0.2 | 0.1 | 0.2 | 0.2 |
| p38 MAPK | 0.1 | 0.1 | 0.3 | 0.2 |
| General transcription regulation | 0.1 | 0.1 | 0.2 | 0.2 |
| Notch signaling | 0.1 | 0.2 | 0.1 | 0.2 |

Abbreviations: EGF: epidermal growth factor, CCKR: cholecystokinin receptor, PDGF: platelet-derived growth factor, TGF: transforming growth factor, FGF: fibroblast growth factor, 5HT: 5-hydroxytryptamine VEGF: vascular endothelial growth factor, GABA: gamma-aminobutyric acid, IGF: insulin-like growth factor, HIF: hypoxia-inducible factor.

1. Zeighami, Y., et al., *Assessment of a prognostic MRI biomarker in early de novo Parkinson's disease.* Neuroimage Clin, 2019. **24**: p. 101986.
